# Socket Preservation Using Dentin Mixed with Xenograft Materials: A Pilot Study

**DOI:** 10.3390/ma16144945

**Published:** 2023-07-11

**Authors:** Elio Minetti, Andrea Palermo, Paolo Savadori, Assunta Patano, Alessio Danilo Inchingolo, Biagio Rapone, Giuseppina Malcangi, Francesco Inchingolo, Gianna Dipalma, Francesco Carlo Tartaglia, Angelo Michele Inchingolo

**Affiliations:** 1 Department of Biomedical, Surgical, and Dental Science, University of Milan, 20122 Milan, Italy; elio.minetti@unimi.it (E.M.); paolo.savadori@unimi.it (P.S.); 2 College of Medicine and Dentistry, Birmingham B4 6BN, UK; andrea.palermo2004@libero.it; 3 Fondazione Ca’Granda IRCCS Ospedale Maggiore Policlinico, 20122 Milan, Italy; 4 Department of Interdisciplinary Medicine, University of Bari “Aldo Moro”, 70121 Bari, Italy; assuntapatano@gmail.com (A.P.); ad.inchingolo@libero.it (A.D.I.); biagiorapone79@gmail.com (B.R.); giuseppinamalcangi@libero.it (G.M.); angeloinchingolo@gmail.com (A.M.I.); 5 Department of Biomedical Sciences, Humanitas University, Via Rita Levi Montalcini 4, 20072 Pieve Emanuele, Italy; francesco.tartaglia@st.hunimed.eu

**Keywords:** socket preservation, dentin matrix, xenograft material, autologous tooth graft, biomaterials, oral surgery

## Abstract

Background: The use of human dentin matrix could serve as an alternative to autologous, allogenic, and xenogeneic bone grafts due to its osteoinductive characteristics. The limitations of its use is tooth availability and that it is often necessary to mix it with a biomaterial. Aim: The aim of this study was to analyze a mix of two different graft materials with different reabsorption ranges when the dentin graft material was not sufficient for full socket preservation. Methods: Seven socket preservation surgeries were carried out employing a mixed graft material containing 50% dentin and 50% xenograft. After four months of recovery, the implants were positioned. At the time of the prosthesis placement and implant surgery, bone samples were collected. Results: The histologic analysis revealed no inflammatory or infective reaction against the seven biopsies. The histomorphometric graft analysis revealed an amount of New Bone of 29.03 ± 6.57% after 4 months and 34.11 ± 5.02% after 8 months. Conclusions: The two graft materials had a different volume reabsorption rate: 71% after 4 months and 90% after 8 months for dentin, and 6% after 4 months and 26% after 8 months for the xenograft. The space created by the dentin reabsorption increased the quantity of new bone.

## 1. Introduction

The possibility of positioning dental implants in sites that have undergone tooth extraction is strictly reliant on the available bone volume that results from the bone healing and remodeling after tooth removal. Bone remodeling is a continuous, complex process that involves all the bone tissues in one organism and can lead to the resorption of alveolar bone when a tooth is no longer present [1]. This process results in a vertical volumetric alveolar bone reduction (1.67 and 2.03 mm) and a horizontal volumetric alveolar bone reduction (3.87 mm) [2].

These variations in soft and hard tissues mainly occur during the first year after extraction [3].

To minimize these problems, a socket preservation technique has been suggested as it helps reduce soft and hard tissue volumetric contractions (0.36 mm horizontal bone loss and 0.58 mm vertical bone loss) [4].

After tooth extraction, the socket undergoes a series of complex healing processes—starting with clot stabilization, fibrin production, and recruitment of osteoblasts, which are responsible for new bone growth. Various biomaterials and procedures with different functionalities—such as osteoconduction and osteoinduction—have been documented to maintain alveolar volume, depending on the characteristics of each material [5].

Several studies have provided histological evidence of new bone formation in extraction sockets following the application of barrier membranes in combination with allogeneic [6] and xenogenic [7] bone grafts.

Guided bone regeneration (GBR) was introduced as a treatment approach with the objective of promoting bone regeneration through the utilization of barrier membranes.

The therapeutic protocol of GBR involves surgically placing a membrane that prevents cell migration, thereby sealing off the area of the bone requiring regeneration.

By creating and sustaining an isolated space, the membrane establishes an environment conducive to the presence and activity of osteoprogenitor cells [8,9].

The graft materials are classified as autograft (bone from the same patient), allograft (bone from another human), dentin (material from the patient’s own teeth), xenograft (bone from another species), and alloplast (synthetic material). Approximately 87% of the graft material market consists of alloplast and xenografts (infodent 12-2018), and their only property is osteoconduction. Most sintered HAP ceramics, including the porous ones, have large crystals that are difficult to remodel. Therefore, residues of graft material can impede the space required for new bone formation, as they are reabsorbed with great difficulty. Placement of the biomaterial in the fresh extraction socket can slow healing. Bio-Oss^®^ particles, for example, are not resorbed, but become surrounded by new bone [10,11,12].

In 2003, Norton found about 26.9% of new bone and 25.6% of xenograft material following remodeling (Bio-Oss) [13].

Autogenous bone has been extensively used due to its osseoinductive, osseoconductive, and osteogenic properties [14,15]. However, the high resorption rate, donor site morbidity, and limited availability can compromise the clinical outcomes of autogenous bone grafts [16,17].

Xenografts are the most commonly used osteoconductive graft material, and Corbella et al. evaluated 273 biopsies from 14 studies [18,19,20].

The use of dentin matrix from autologous extracted teeth could be an alternative, promoting bone healing in intraoral defects [21,22,23,24,25,26,27].

Urist et al. discovered the osteoinduction potential of demineralized dentin matrix over 50 years ago, when they invented the tooth-grafting process [28,29]. More recently, Bessho et al. demonstrated the existence of bone morphogenetic proteins (BMPs) in the matrix of human dentin. In particular, ectopic bone formation and the presence of osteoblasts were observed in rat muscle after demineralized human dentin matrix grafting [30].

Dentin should be considered a graft material with osteoinductive and osteoconductive properties due to the presence of numerous proteins shared with bone within it [31,32,33]. Recent human studies have shown the effectiveness and safety of chairside-prepared autogenous partially demineralized dentin matrix for clinical applications in bone regeneration procedures related to implant dentistry, including alveolar ridge augmentation, socket preservation, and maxillary sinus floor augmentation [33,34,35,36,37,38,39].

Several experimental findings from a thorough literature analysis show how demineralized dentin matrix affects osteodifferentiation in vitro and increases osteoinduction in vivo. Some authors theorize that the demineralization process allows for better bone augmentation compared to non-demineralized dentin—likely because the availability of these molecules increases with matrix demineralization [32,40,41].

The chemical composition of bone and dentin is quite similar, consisting of an inorganic portion made of hydroxyapatite and an organic portion mainly composed of collagen type 1 and other secondary proteins [42,43,44].

The proteins in the extracellular matrix (ECM) of bone and tooth are better protected. The apatite in which the proteins are embedded apparently provides significant protection against postmortem destruction caused by chemical and physical agents. As a result, these structures can be preserved for many thousands of years after death [45]. Demineralized dentin’s capability to promote bone regeneration is highly comparable to the promotion of new bone growth by bone autografts. These mechanisms are characterized by cellular differentiation processes triggered by the interaction of cells that stimulate bone growth and cells that respond to it [31,32,33].

An innovative medical device capable of processing extracted teeth and producing a suitable graft material has been introduced to the market. This system guarantees fully automated demineralization, grinding, and cleaning procedures, eliminating the need for human intervention in the process. According to some researchers, the mineralized allograft is only claimed to be osteoconductive; however, when the material is resorbed and remodeled throughout the bone regeneration process, osteoinductive elements within the mineralized structure may also become accessible [46,47,48].

However, it can be challenging to fill large bone defects using only demineralized dentin, due to its limited availability. Therefore, in a study conducted by Umebayashy et al., they suggested combining different materials when dentin alone is insufficient. Specifically, they described a case where bilateral alveolar bone and sinus floor augmentation were achieved by grafting a mixture of demineralized dentin, cancellous bone particles, and medullary bone particles, followed by full-arch implant rehabilitation. No complications occurred after 4 years, and both the bone volume and physiological bone volume were successfully maintained [49].

Another study examined the use of bone marrow stromal cells engineered to express the dentin matrix protein-1 gene, in combination with Bio-Oss(^®^), for sinus augmentation with simultaneous implant placement in dogs. Bone regeneration and osseointegration were evaluated using histologic and histomorphometric methods after a three-month healing period. The combination of materials demonstrated osteoinductive and osteoconductive bone regeneration capacities [50].

The primary goal of the study was to investigate situations where there was limited tooth structure available for regeneration, requiring the utilization of grafting materials, and to evaluate the effectiveness of this approach and determine if it demonstrated any favorable attributes.

Based on the Norton considerations [13], this study aimed to observe and compare the graft reabsorption curve, measured through histomorphometry after 4 months and 8 months. The goal was to determine if it is possible to mix two different graft materials with varying reabsorption ranges when the dentin graft material alone is insufficient to fill the entire socket preservation. The study aimed to take advantage of dentin reabsorption in such cases.

## 2. Materials and Methods

### 2.1. Study Design and Patient Selection

Seven subjects (3 men and 4 women) with a mean age at surgery of 53 ± 67.89 years (ranging from 42 to 63 years) were enrolled and treated following the described protocol. All patients considered for inclusion in the study were in good health and treated in three dental clinics located in Italy.

The sample size of this study consisted of 7 cases after 4 months and the same 7 cases after 8 months. Experienced operators performed the surgical procedures and all patients provided written informed consent prior to participating in the study. One autologous tooth (extracted due to cavity or periodontal disease), either element 4.6 or 3.6, was extracted from each patient and used to prepare a tooth graft. The tooth graft was mixed with 50% xenograft material and utilized using socket preservation techniques. All socket preservations involved three-wall defects. The graft was covered with an absorbable membrane (Osseoguard, Zimmer, Collagen matrix, Inc., Okland, NJ, USA).

The marginal gingival tissue was slightly, gently stretched to cover the alveolus and closed by single sutures. The socket was completely filled with the graft.

The primary objective of this prospective study was to analyze the histological differences and evolutions between the two graft materials used for socket preservation. To achieve this goal, two histological samples were taken: the first after 4 months of regeneration and the second after 8 months of regeneration.

Following the operation of the TT Tooth Transformer device, a combination of 50% autologous tooth and 50% xenograft (Bio-Oss, Geistlich Pharma AG, Wolhusen, Switzerland) was used to preserve the sockets of 7 patients after tooth extraction.

**Figure 1:**
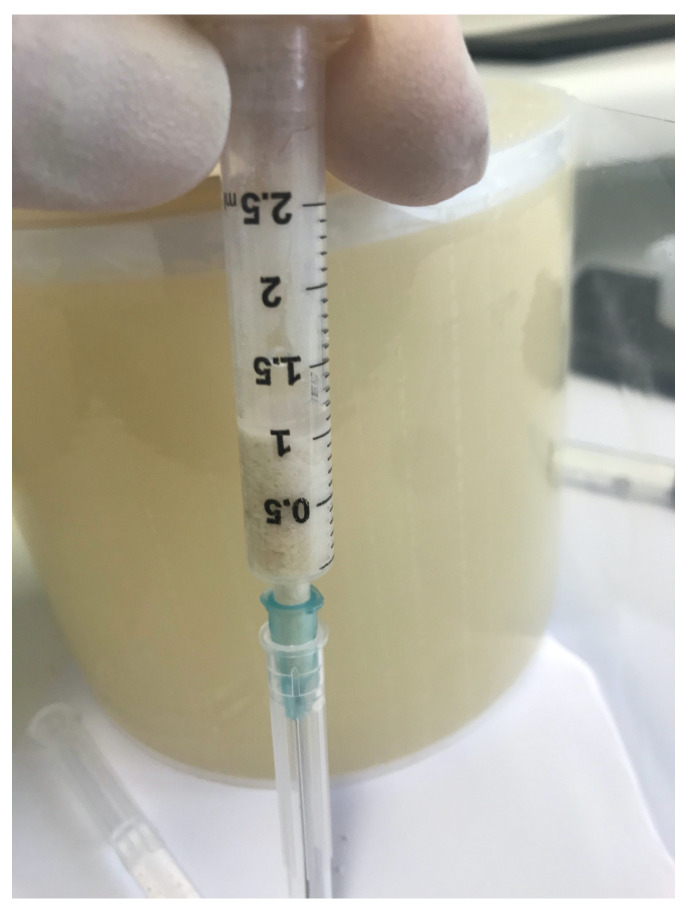
The syringe containing 1 cc of granules and demineralized water inserted drop by drop.

To understand the volume occupied by each individual graft material, a test was conducted. The test involved loading a defined value of granules (1 cc) into a syringe. Drop by drop, demineralized water was introduced until the volume of the granules was reached. The quantity of water used represented the measure of the volume occupied by the space between the granules. This method allowed us to understand the percentage change in the material over time through histomorphometric values (Figure 1).

### 2.2. Inclusion Criteria

The general inclusion criteria were those commonly adopted in the three clinics: patients aged at least 18 years, in good general health (ASA-1 and ASA-2), and able to undergo surgical and restorative procedures. Specific criteria included patients needing socket preservation after tooth extraction to maintain the required bone volume for implant insertion. The reasons for extraction had to be periodontal disease, fractures, or caries. The study group comprised seven patients with seven sites requiring three-wall socket preservation.

### 2.3. Exclusion Criteria

Patients with a history of tobacco use within the last 6 months, allergies, diabetes, healing disorders, HIV, cancer, metabolic diseases, bone diseases, systemic corticosteroid use, intravenous or intramuscular bisphosphonate use, immunosuppressive agent use, radiation therapy, or chemotherapy (within the last 2 months) were excluded. Pregnant women were also excluded. These exclusion criteria were chosen to select cases that may not be influenced by any underlying pathologies.

### 2.4. Surgical Procedures and Follow-Up

Histological and morphometrical data were detected in accordance with the protocol recorded at the University of Chieti (Ethical Committee approval: request ID richhtnc4, protocol N_1869 12 December 2018, approved 17 verb 21.03.19 St.638 PI Perfetti). Extractions were performed after administering antibiotics. Bone defects were filled using the following technique: The tooth was first thoroughly cleansed of any remaining calculus, then the root surface was polished with a diamond drill and ample irrigation. Previous fillings, such as composite or gutta-percha, were removed. The tooth was divided into small pieces and placed inside the grinder (Tooth Transformer TT, Milan, Italy). The single-use device was opened, and a small package of disposable liquids was placed in the designated location. The company claims that these treatments provide both root decontamination and maximal release of embedded proteins. Once all the parts were installed and the machine’s cover was closed, the general switch button was used to turn on the device. After 25 min, the demineralized dentin graft was ready to be introduced into the patient’s mouth (TT TOOTH TRANSFORMER SRL, Milan, Italy), along with Xenograft (Bio-Oss Geistlich Pharma AG) in a 50% proportion. An absorbable collagen membrane (Osseoguard–Zimmer) was used to cover the grafts and extend approximately 2–3 mm beyond the borders of the bone defect. Amoxicillin was administered in doses of 1000 mg every 12 h, or 2000 mg BID for 10 to 14 days. One week after the procedure, the sutures were removed. After four months of healing, biopsies were performed during implant placement using a trephine cylindrical drill with graduated depth markings (ranging from 5 to 18 mm) and abundant sterile saline irrigation. After another 4 months, during the implant healing period or healing screw insertion, another biopsy was performed using a smaller diameter trephine cylindrical drill with graduated depth markings (ranging from 5 to 18 mm) under copious sterile saline irrigation.

### 2.5. Collection and Statistical Analysis of Data

Records of patients, including histological and morphometric data, were collected and stored in separate files, in compliance with privacy legislation. To establish accurate reference values for comparison with other studies and between the two groups, the average values of the morphometric data were calculated.

### 2.6. Histological Technique

Histomorphometric analysis was conducted on both decalcified and non-decalcified bone samples. The bone quantification procedure was consistent for both types of biopsies, ensuring that the quantification of new bone did not significantly impact data collection [51,52].

- Decalcified:

The samples were sliced, embedded in paraffin, and subjected to decalcification. The samples were preserved in 10% neutral buffered formalin for 7 days, consisting of a solution of 37% formaldehyde, 10 mL of NaCl, 0.8 g of potassium phosphate monobasic, 0.65 g of potassium phosphate dibasic, and 90 mL of distilled water. Disodium EDTA pH 7 was used for decalcification until complete decalcification was achieved, determined by physical examination. Subsequently, the samples were dehydrated in ethanol with increasing concentrations from 70% to 100%, cleaned with xylene, and embedded in paraffin using Carlo Erba reagents. Paraffin slides were cut using the Lecia RM2245 (Leica Biosystems, Milan, Italy) rotatory microtome and mounted on superfrost microscope glass slides with Biomount HM (Bio-Optica Milano Spa, Milan, Italy). Histology images obtained from the Olympus transmitted light microscope were converted to digital photographs using a digital camera and analyzed using the image analysis program IAS 2000 (QEA). The following histomorphometric measurements were calculated for each sample:

BV%: Percentage of mineralized tissue excluding medullary tissues.
Graft%: Percentage of the volume occupied by the remaining graft or dentin.
VB%: Percentage of vital bone excluding medullary tissues. The sum of TT% and VB% represented the BV%. Each subsection was measured using the ImageJ 1.52q program.

- Non-decalcified:

Each sample underwent dehydration using a series of alcohol solutions with increasing concentrations until reaching pure alcohol, followed by infiltration with methacrylic resin. After the resin was light-cured, non-decalcified sections were obtained using a disk abrasion system (LS2-Remet, Bologna, Italy) and a diamond disk cutting system (Micromet-Remet, Bologna, Italy). The region of interest in the biopsy was exposed by removing the resin component covering the sample through resin abrading. The exposed surface was then attached to a showcase using cyanoacrylate adhesives. Subsequently, the sample was cut with a high-speed diamond blade under cooling conditions to obtain a thickness of approximately 200 μm, which was further thinned by abrasion to about 40–50 μm. The slide was polished with polishing papers and stained with basic fuchsin and blue toluidine for final observation under light and polarized light microscopy.

For histomorphometric measurements, the histological images obtained from the transmitted light microscope were digitized through a digital camera and analyzed by means of image analysis software IAS 2000.

For each sample, the following were calculated using histomorphometric analysis:

BV% = Percentage of residual bone volume, excluding medullary tissues;
Graft% = Percentage of the remaining graft, without bone and marrow;
VB% = Percentage of vital bone, excluding the bone marrow and the residual graft.

## 3. Results

**Figure 2:**
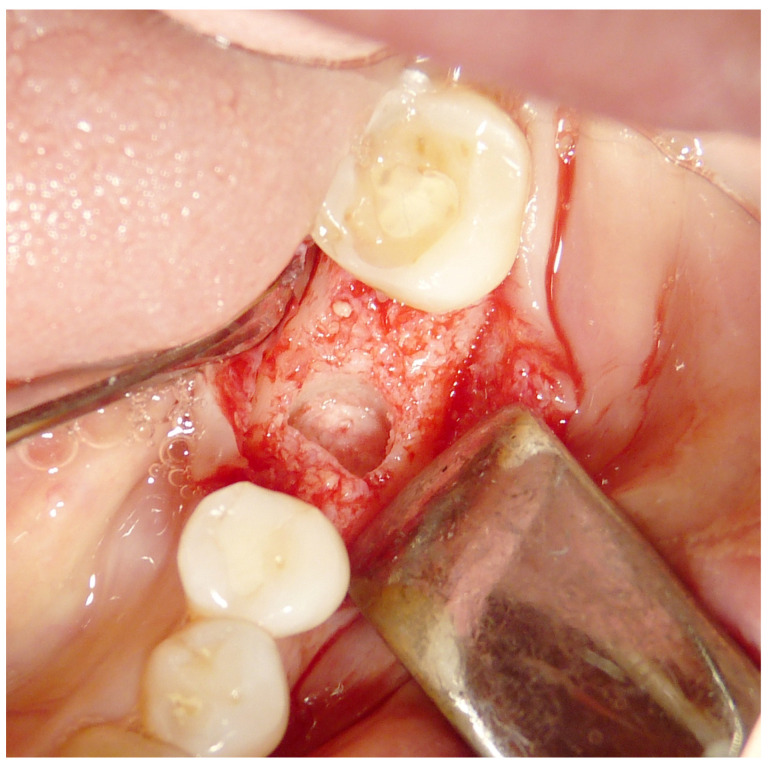
First histology after 4 months healing.

**Figure 3:**
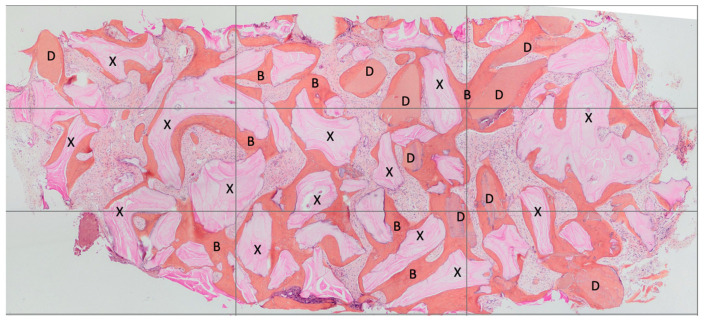
Histology from 4 months healing In this histological image, the remaining xenograft material granules can be observed—marked with X. The residual dentin granules are marked with D. The bone tissue is highlighted with B. It is evident, both visually and as supported by histomorphometry, that the xenograft material granules were less resorbed compared to the dentin granules.

**Figure 4:**
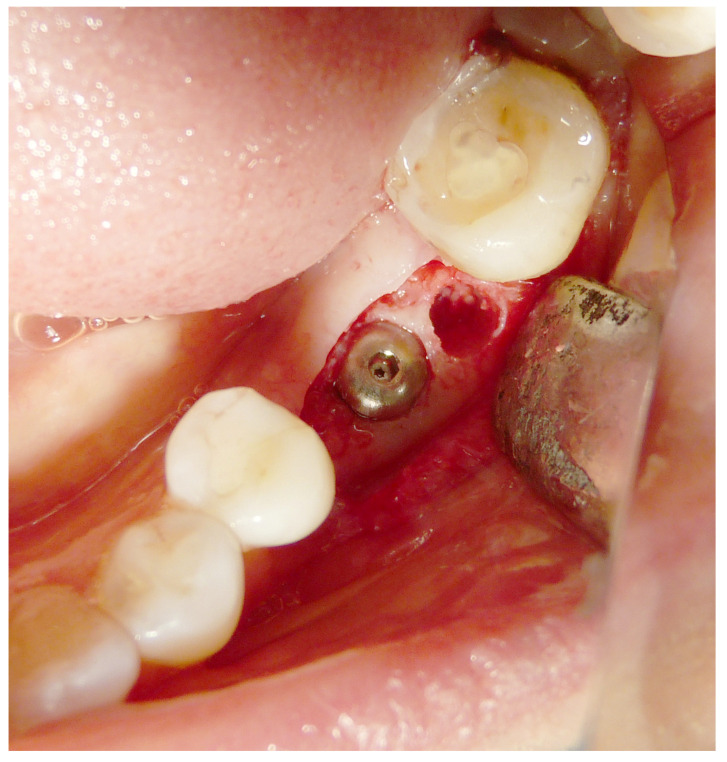
Second histology after 8 months of healing.

**Figure 5:**
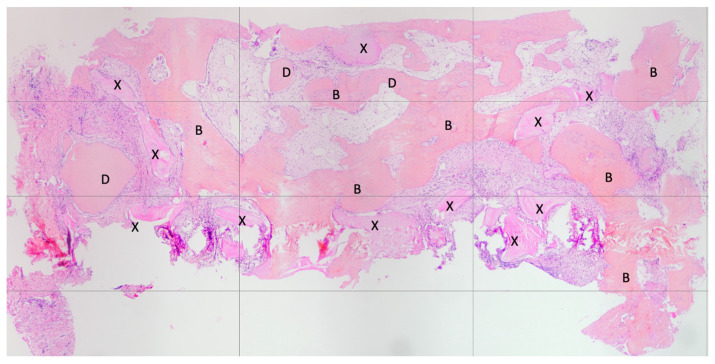
Histology after 8 months from the same patient. In this histological image, the remaining xenograft material granules can be observed—marked with X. The residual dentin granules are marked with D. The bone tissue is highlighted with B. It is evident, both visually and as supported by histomorphometry, that the xenograft material granules were less resorbed compared to the dentin granules.

Both hard and soft tissues remained steady throughout the healing process. There were no difficulties with the soft tissue grafting treatments’ healing. After a healing period of 4 months, seven biopsies (Figure 2 and Figure 3) were performed during the implant insertion procedure; after the healing period (8 months after the socket preservation procedure), seven biopsies were performed (Figure 4 and Figure 5).

The results of the histomorphometric analysis of the graft biopsies are presented in Table 1. The syringe test indicated that approximately 60% of the space was occupied by the graft, while the remaining 40% represented the space between the granules (Table 2).

Initially, the graft composition was 30% dentin graft and 30% Bio-Oss, accounting for 60% of the total space occupied by the combined graft. The remaining 40% represented the space between the individual graft granules.

**Figure 6:**
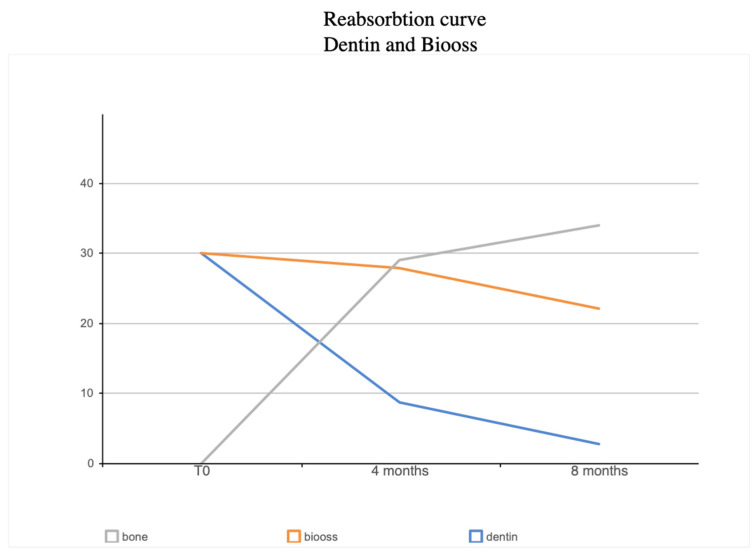
This graph illustrates the different rates of resorption of the two materials, observed through histomorphometry at 4 months and 8 months. Initially, at time zero, both the dentin and Bio-Oss grafting materials occupied 60% of the space, with no newly formed tissue present.

Over time, the graft percentages changed significantly. After 4 months, the volume percentage of the dentin graft decreased by 71%, while the Bio-Oss graft only decreased by 6.74%. After 8 months, the dentin graft volume percentage decreased by 90.71%, while the Bio-Oss graft volume percentage decreased by 26.42%. Figure 6 highlights an interesting inverse relationship between the dentin curve and the new bone curve. It can be observed that the BV% (Bone Volume %) decreased over time, while the NB% (New Bone %) increased. This can be attributed to the reabsorption of the graft material, and demonstrates the relationship between the remaining graft quantity and the rate of reabsorption.

At four months, the dentin (blue line) showed partial resorption, measuring approximately 8%. In contrast, the Bio-Oss material (red line) exhibited less pronounced resorption, with approximately 28% remaining. The gray line representing the bone tissue showed an increase of 29%.

By eight months, the trend in dentin resorption persisted, and the blue line dropped to around 2%—indicating that 90.71% of the dentin had been resorbed. The resorption of Bio-Oss, represented by the red line, remained at approximately 22.9%—indicating that only 26% of the Bio-Oss material had been resorbed. Meanwhile, the bone tissue, benefiting from the resorption of the two biomaterials, had grown within the spaces and accounted for 34% of resorption (gray line).

## 4. Discussion

The main objective of this study was to investigate cases where a minimal tooth structure existed for regeneration, requiring the addition of grafting material. The aim was to assess the effectiveness of this approach and determine if it demonstrated any desirable qualities.

Numerous clinical investigations have examined the healing mechanisms of post-extraction sites following bone grafting procedures, focusing on various graft materials [18,53,54,55].

Recent systematic reviews and meta-analyses have shown that xenogeneic materials generally perform well. Surgeries utilizing allografts have the lowest percentages of remaining graft material (12.4–21.11%), while those using xenografts and alloplasts yield better results after 7 months (37.14% and 37.23%) [18].

From a therapeutic perspective, it has been suggested that this family of grafts may reduce tridimensional shrinkage of the bony crest if sealed with a collagen membrane. However, from a histological perspective, these materials could potentially result in incomplete resorption due to their composition. The bone regeneration scaffold should provide structural support for bone development, possess mechanical qualities similar to the host bone, be biocompatible, and degrade at a rate compatible with bone remodeling [18].

The use of tooth grafts for socket preservation has been described in different studies. Kim et al. suggest utilizing autogenous fresh demineralized tooth grafts created chairside immediately after extraction. Radiographs reveal good bony healing, while histologic sections have demonstrated appropriate new bone development and resorption patterns [56]. Minetti et al. employed demineralized dentin matrix derived from vital or endodontically treated teeth in combination with a collagen membrane for post-extraction site preservation [36].

This study builds upon considerations made by Lindhe and Norton, who observed that the placement of Bio-Oss or non-resorbable graft materials in fresh extraction sockets delayed healing, as the particles were not resorbed, but instead were surrounded by new bone [10,57,58,59].

A total of 25 patients undergoing single-tooth extraction and implant placement were included in the study. They were divided into two groups: the test group, in which the socket was filled with Bio-Oss collagen (Geistlich Pharma, Wolhusen, Switzerland) and covered with a collagen membrane (Mucograph, Geistlich Pharma), and the control group, in which the socket was filled with a blood clot and covered with a collagen membrane (Mucograph, Geistlich Pharma). After six months of healing, non-inflamed tissues were observed in both test and control sites. However, in the control sites, cortical bone was present, whereas in the Bio-Oss collagen-grafted sites, distinct cortical bone was lacking; histological analysis revealed compromised central and apical regions, with the biomaterial surrounded by connective tissue [45].

The average amount of new bone indicated in the study was 26.9% and 25.6% for the xenograft material (Bio-Oss) [13]; this can be attributed to the phagocytic abilities of osteoclasts, the bone-resorbing cells, and the low pH they create in their surroundings, leading to the dissolution of hydroxyapatite (HAP) ceramics upon contact with mineral cells [60].

The challenge arises from the fact that most sintered HAP ceramics—even the porous ones—have large crystals that are difficult to remodel [61].

Some authors suggest that the presence of non-resorbable graft materials makes it difficult for new bone to grow, as the space is occupied by the graft [10].

In contrast, dentin—after appropriate treatment—undergoes almost complete resorption. In a recent histological publication, the effectiveness of dentin as a graft in alveolar socket preservation (ASP) procedures was evaluated through the first prospective histomorphometric evaluation. A total of 101 histologies from 96 subjects (50 females and 46 males) with a mean age of 56.3 ± 14.7 years were analyzed after guided bone regeneration (GBR) procedures using the ToothTransformer treatment. After an average healing time of 5.2 ± 1.9 months, the residual graft amounted to 7.7 ± 12.2% in the maxilla and 7.0 ± 11.1% in the mandible [62].

Therefore, in cases where there is a reduced amount of dentin—insufficient for proper socket preservation—the addition of a reabsorbable biomaterial to Bio-Oss or a non-resorbable graft material may have advantages. This is due to the resorption of dentin, creating space for new bone to form.

Table 1 illustrates that the Bone Volume % (BV%) and New Bone % (NB%) have an inverse relationship over time. This is attributed to the resorption of the graft material, and the relationship between the remaining graft quantity and the rate of reabsorption can be understood.

In Table 1, it can be observed that the BV% remained at around 60% (65% at 4 months and 59% at 8 months), indicating that the volume was maintained over time—but the distribution of the volume changed. Initially, the graft was composed of 30% dentin graft and 30% Bio-Oss—occupying 60% of the space, with the remaining 40% representing the space between individual graft granules. Over time, the graft percentages exhibited significant differences: the dentin graft volume decreased by 71%, while the Bio-Oss graft volume only decreased by 6.74%. After 8 months, the dentin graft volume % decreased by 90.71%, and the Bio-Oss graft volume % decreased by 26.42%. Table 2 highlights the interesting inverse similarity between the dentin curve and the new bone curve.

The histological results of the treated dentin with TT (ToothTransformer) were comparable to a recent publication that analyzed 101 histologies after guided bone regeneration (GBR) procedures using TT [62].

## 5. Conclusions

The present study revealed different rates of reabsorption between two distinct graft materials during the healing process. It is interesting to note that the space created by dentin reabsorption contributes to an increased quantity of new bone compared to the insertion of xenograft material alone.

This study aimed to address the issue of the limited availability of natural tooth material for grafting and the need to supplement it with alternative materials from different sources. This question has remained unanswered in the scientific community until now. Despite the limitations in sample size, we endeavored to provide an answer to this inquiry. Undoubtedly, the use of a resorbable grafting material in combination with a partially resorbable material ensures superior outcomes compared to the use of only a partially resorbable material; this is because the space occupied by the graft granules becomes readily available for new bone formation, while the structure is maintained by the non-resorbed granules.

Given the limitations in the number of samples and the constraints of the histomorphometric analysis (such as the dimensions and location of the biopsy), we recommend conducting further studies—particularly randomized controlled clinical trials with larger sample sizes—to confirm the results already obtained in a significant number of pilot studies.

## Figures and Tables

**Table 1:** The mean histomorphometry of the samples shows the percentage of graft loss relative to the initial quantity of graft inserted in the socket preservations. This table presents the changes in the percentages of dentin, Bio-Oss, and bone over time, as determined by histomorphometry. Additionally, a separate table illustrates the percentage resorption rate of the two materials for comparison.

	BV%	NB%	Residual Graft Dentin %	Dentin Loss Percentage	Residual Graft Bio-Oss %	Bio-Oss Loss Percentage
4 Months	65.66%	29.03± 6.57%	8.68 ± 3.36%	71%	27.95 ± 5.23%	6.74%
8 Months	59%	34.12 ± 5.05	2.79 ± 1.48	90.71%	22.09 ± 13.81	26.42%

**Table 2:** Percentage of space occupied by granules and water.

	Granules	Water
Percentage of space 100%	60%	40%

## Data Availability

Not applicable.

## Abbreviations

BMPs	Bone Morphogenetic Proteins
ECM	Extracellular Matrix
TT	Tooth Transformer
BV	Bone Volume
NB	New Bone
